# Nitrogen starvation-induced cellular crosstalk of ROS-scavenging antioxidants and phytohormone enhanced the biofuel potential of green microalga *Acutodesmus dimorphus*

**DOI:** 10.1186/s13068-017-0747-7

**Published:** 2017-03-09

**Authors:** Kaumeel Chokshi, Imran Pancha, Arup Ghosh, Sandhya Mishra

**Affiliations:** 1 Division of Salt & Marine Chemicals, CSIR-Central Salt and Marine Chemicals Research Institute, Bhavnagar, Gujarat 364002 India; 2 Academy of Scientific & Innovative Research (AcSIR), CSIR-Central Salt and Marine Chemicals Research Institute, Bhavnagar, Gujarat 364002 India; 3 Division of Plant Omics, CSIR-Central Salt and Marine Chemicals Research Institute, Bhavnagar, Gujarat 364002 India; 4 Laboratory for Chemistry and Life Science, Institute of Innovative Research, Tokyo Institute of Technology, Yokohama, 226-8503 Japan

**Keywords:** Microalgae, Biofuel, Nitrogen starvation, Lipid, Oxidative stress, Reactive oxygen species, Stress-responsive hormone

## Abstract

**Background:**

Microalgae accumulate a considerable amount of lipids and carbohydrate under nutrient-deficient conditions, which makes them one of the promising sustainable resources for biofuel production. In the present study, to obtain the biomass with higher lipid and carbohydrate contents, we implemented a short-term nitrogen starvation of 1, 2, and 3 days in a green microalga *Acutodesmus dimorphus*. Few recent reports suggest that oxidative stress-tolerant microalgae are highly efficient for biofuel production. To study the role of oxidative stress due to nitrogen deficiency, responses of various stress biomarkers like reactive oxygen species (ROS), cellular enzymatic antioxidants superoxide dismutase (SOD), catalase (CAT), ascorbate peroxidase (APX), and non-enzymatic scavengers proline and polyphenols were also evaluated. Further, the endogenous levels of phytohormones abscisic acid (ABA) and indole-3-acetic acid (IAA) were also determined to study their response to nitrogen deficiency.

**Results:**

We observed that nitrogen starvation of 2 days is effective to produce biomass containing 29.92% of lipid (comprising about 75% of neutral lipid) and 34.80% of carbohydrate, which is significantly higher (about 23 and 64%, respectively) than that of the control culture. Among all nitrogen-starved cultures, the accumulations of ROS were lower in 2 days starved culture, which can be linked with the several folds higher activities of SOD and CAT in this culture. The accumulations of proline and total polyphenols were also significantly higher (about 4.7- and 1.7-folds, respectively, than that of the control) in 2 days nitrogen-starved culture. The levels of phytohormones once decreased significantly after 1 day, increased continuously up to 3 days of nitrogen starvation.

**Conclusion:**

The findings of the present study highlight the interaction of nitrogen starvation-induced oxidative stress with the signaling involved in the growth and development of microalga. The study presents a comprehensive picture of the adaptive mechanisms of the cells from a physiological perspective along with providing the strategy to improve the biofuel potential of *A. dimorphus* through a short-term nitrogen starvation.

## Background

Microalgae are considered among one of the alternative renewable energy feedstocks to reduce our dependency on existing fossil-based fuels [1]. Microalgal lipids and carbohydrates can be used to produce biodiesel and bioethanol, respectively. Till date, microalgae-based biofuels are not fully considered to be economically feasible due to the lower productivities of microalgal biomass, lipid, and carbohydrate [2]. Various reports suggest that accumulations of lipid and carbohydrate by microalgae could be triggered by environmental stress conditions. This includes physical stimuli like changes in culture pH, temperature, light intensity, photoperiod [3, 4], and chemical stimuli like deprivation of nutrients (nitrogen and phosphorous), salinity stress [5]. Among all, nitrogen starvation is considered as a prominent strategy due to its high efficiency [2, 6].

Nitrogen is one of the most important growth elements as it is a major component of the biological macromolecules like DNA, chlorophyll, and protein. Although continuous nitrogen starvation increases lipid and carbohydrate contents of microalgae, it decreases their growth rate, consequently reducing their overall productivities [5]. To overcome such problem, two-stage cultivation strategy is recently becoming popular in which microalgae are first grown in the nutrient-rich medium to achieve the high cell biomass and then subsequently transferred to a nutrient-deficient medium to improve the accumulation of lipid and carbohydrate [7]. However, the strategy and the magnitude of the required nitrogen deficiency differ from microalgal species.

Redox reactions of reactive oxygen species (ROS) like hydrogen peroxide (H_2_O_2_), superoxide (O_2_^·−^), and hydroxyl (OH^·^) radicals with the cellular lipids, proteins, DNA, result in toxicity due to oxidative stress conditions [8]. These ROS are also considered as an important factor in the cellular response. The stress-induced ROS accumulation is counteracted by an integral defense mechanism of the cells, which scavenges the excess oxidants and avoids the harmful effects of ROS. This includes an array of the enzymatic scavengers such as superoxide dismutase (SOD), catalase (CAT), ascorbate peroxidase (APX), and the non-enzymatic antioxidants such as pigments, polysaccharides, polyphenols, proline, carotenoids, and flavonoids [9]. These markers of the oxidative stress are now frequently detected and linked with the lipid accumulation by microalgae cultivated under abiotic stress like temperature [4, 10], salinity [11], nitrogen [12, 13], and light [14].

ROS are also known to interact with other signaling pathways of the plants like those of nitric oxide, calcium, and hormones [15, 16]. Plant hormones (phytohormones), a class of signal molecules that are produced in extremely low concentrations, serve as chemical messengers to coordinate various cellular activities in the higher plants. It has been suggested that hormone systems in higher plants are evolved from a pre-existing primary metabolic system in microalgae [17]. Although basic physiological activities of various phytohormones in higher plants are well described, the evidence of their role in microalgae, particularly in the stress tolerance and their response to ROS accumulation, is fragmentary.

*Acutodesmus dimorphus* is the thermotolerant green microalga [18], a potential strain for biofuel production [4, 19]. In the present study, effects of short-term nitrogen starvation on the morphology and physiology of *A. dimorphus* were evaluated. Changes in the photosynthetic pigments, contents of lipid, carbohydrate, protein, and responses of phytohormones, abscisic acid (ABA), and indole-3-acetic acid (IAA), were determined. The accumulations of ROS and responses of various cellular antioxidants viz. proline, total polyphenols, SOD, CAT, and APX were also evaluated to study the role of oxidative stress in the accumulation of lipid and carbohydrate by *A. dimorphus* under nitrogen deficiency.

## Methods

### Experimental conditions

The experiment was carried out in triplicate in 1 l Erlenmeyer flasks containing 500 ml of BG-11 culture medium inoculated with 10% of the actively growing culture of *A. dimorphus*. Cells were cultivated at 35 °C under 150 µmol/m^2^/s of light intensity (cool white fluorescent lights were used as the light source) and 12:12 h of light: dark period. Flasks were manually shaken thrice a day to avoid the adherence of the cells to the surface of the flasks. After 12, 13, and 14 days of cultivation, cells were harvested by centrifugation at 14,330×*g* for 5 min, washed twice with distilled water, re-inoculated in the nitrate-free BG-11 medium, and cultivated for total 15 days to deliver nitrogen starvation of 3, 2, and 1 days, respectively. The culture grown in the BG-11 medium for 15 days was used as a control.

Morphological changes in the cells were observed using an inverted microscope (Leica DM IL LED, Leica Microsystems). The culture was pipetted onto a clean glass microscope slide and covered with a coverslip. The slide was then placed on the platform and images were obtained using a 40× objective. The cell dimensions were measured using the software Leica application suite, Leica Microsystems.

### Determination of microalgal growth

To determine the dry cell weight (DCW) of the culture after 15 days of cultivation, a known volume of culture was collected in moisture free pre-weighed centrifuge tubes and centrifuged at 14,330×*g* for 5 min. The supernatant was discarded and the tubes were dried in an oven at 60 °C until constant weight. The tubes were then transferred to the desiccator, cooled down to room temperature, and the post-weight was recorded. The DCW was determined by calculating the difference in the weights of the tubes and expressed in mg/l.

### Analysis of pigments content

For the analysis of pigments content, 2 ml culture was centrifuged at 8270×*g* for 5 min, the supernatant was discarded and 2 ml of 99.9% methanol was added to the pellet. The content was mixed properly and incubated at 45 °C for 24 h in the dark. The extracts were then centrifuged at 8270×*g* for 5 min; the absorbances of the supernatant were read at 470, 652.4, and 665.2 nm and corrected for the turbidity by subtracting the absorbance at 750 nm. The pigments contents were calculated using the following equations [20]:

$${\text{Chlorophyll }}a ; {\text{Chl-}}a \left( {\upmu {\text{g/ml}}} \right) = 16.72 (A_{665.2} ) - 9.16 (A_{652.4} )$$

$${\text{Chlorophyll }}b ; {\text{Chl-}}b ( {\upmu {\text{g/ml}}} ) = 34.09 (A_{652. 4} ) - 15.28 (A_{665.2} )$$

$${\text{Carotenoids }} ( {\upmu {\text{g/ml}}} ) = \frac{{ 1000 (A_{470} ) - 1.63 \left( {{\text{Chl-}}a} \right) - 104. 9 ( {\text{Chl-}}b )}}{221}$$

The calculated pigments contents were converted to a dry weight basis and presented in mg/g.

### Measurement of quantum yield of photosystem II

The maximum quantum yield (*F*_v_ */F*_m_) of photosystem II (PS II) was measured with pulse-amplitude-modulated (PAM) fluorometry (AquaPen-C AP-C100, Photon System Instruments). All cultures were appropriately diluted to an identical absorbance (optical density at 750 nm). The fluorescence of the samples was measured according to the manufacturer’s manual after dark adapting the cells for 20 min. The dark-adapted minimum level of fluorescence (*F*_0_) and the maximum level of fluorescence measured after a short light pulse of high intensity (*F*_m_) were used to calculate *F*_v_ */F*_m_ according to the following equation:

$$F_{\text{v}} /F_{\text{m}} = ( {F_{\text{m}} -F_{ 0} } ) /F_{\text{m}}.$$

### Determination of lipid, carbohydrate, and crude protein contents

The lipid was extracted from the dried microalgal biomass using chloroform:methanol (1:2, *v/v*) [21]. Approximately 200 mg of biomass was mixed with 30 ml of the solvent and ultrasonicated for 2 min (4–5 cycles) at an ambient temperature. The content was centrifuged at 12,860×*g* for 5 min; the supernatant was filtered and transferred to a pre-weighed glass beaker. The solvent was evaporated at 60 °C in an oven, the lipid content was determined gravimetrically and expressed on a dry weight (DW) basis. Total lipid was further fractionated by silica gel (60–120 mesh) column chromatography [22] using chloroform:acetic acid (9:1, *v/v*) to collect neutral lipids (NL), acetone:methanol (9:1, *v/v*) to collect glycolipids (GL) and methanol to collect phospholipids (PL).

For the determination of carbohydrate content, a known quantity of dried microalgal biomass was digested using 72% (*w/w*) sulfuric acid by incubating at room temperature for 1 h [23]. The hydrolysate was diluted with the distilled water to reduce the sulphuric acid concentration to 4% (*w/w*) and the content was incubated at 121 °C for 1 h. The content was cooled to room temperature and total volume was made up to 50 ml with the distilled water. The content was centrifuged at 12,860×*g* for 5 min and the supernatant was used to determine total sugar content by phenol sulphuric acid method [24].

For the determination of the crude protein content, total nitrogen content of the dried microalgal biomass was measured using a CHNS elemental analyzer (Perkin-Elmer Model 2400, USA) calibrated using acetanilide as a reference standard. The crude protein content was calculated using the nitrogen-to-protein conversion factor of 6.25 [25].

### Level of lipid peroxidation

Lipid peroxidation was determined in terms of malondialdehyde (MDA) content in the cells [26]. Microalgal cells were harvested by centrifugation, homogenized in 2 ml of 80:20 (*v:v*) ethanol:water followed by centrifugation at 15,880×*g* for 10 min. An aliquot of 1 ml of the supernatant was mixed with 1 ml of thiobarbituric acid (TBA) solution comprising 20.0% (*w/v*) trichloroacetic acid (TCA), 0.01% butylated hydroxytoluene, and 0.65% TBA. Samples were then mixed vigorously, heated at 95 °C for 25 min, and cooled. The contents were centrifuged at 15,880×*g* for 10 min and absorbances of the supernatants were read at 450, 532, and 600 nm. The MDA content was calculated using the following formula and expressed on a fresh weight (FW) basis:

$${\text{MDA (}}\upmu {\text{mol/g FW) = }}\frac{{ [ 6.45 \times (A_{532} -A_{600} ) ] - [0.56 \times A_{450} ]}}{\text{FW }}.$$

### Measurement of ROS

For the measurement of H_2_O_2_ content, microalgal cells were harvested by centrifugation and the cell pellet was homogenized in 0.1% *w/v* TCA solution. The homogenate was centrifuged at 15,880×*g* for 10 min. An aliquot of 0.5 ml of the supernatant was mixed with 0.5 ml of 10 mM phosphate buffer (pH 7.0) and 1 ml of 1 M potassium iodide. The absorbance of the solution was read at 390 nm [27]. The H_2_O_2_ concentration (μmol H_2_O_2_/g FW) in the sample was determined from a calibration curve prepared using the known concentrations of H_2_O_2_.

For the measurement of O_2_^·−^ content, microalgal cells were harvested by centrifugation, homogenized with 5 ml of 65 mM potassium phosphate buffer (pH 7.8), and centrifuged at 14,330×*g* for 5 min. An aliquot of 1 ml of the supernatant was mixed with 0.9 ml of 65 mM potassium phosphate buffer (pH 7.8) and 0.1 ml of 10 mM hydroxyl ammonium chloride. After incubation at 25 °C for 20 min, 1 ml of 17 mM sulphanilic acid, and 1 ml of 7 mM α-naphthylamine were added to the mixture. After further incubation for 20 min, the absorbance of the solution was read at 530 nm [28]. A standard curve plotted with sodium nitrite was used to calculate the production rate of O_2_^·−^.

For the measurement of OH^·^ content, microalgal cells were harvested by centrifugation, homogenized with 2 ml of 50 mM potassium phosphate buffer (pH 7.0), and centrifuged at 14,330×*g* for 5 min. Thereafter, 0.5 ml of supernatant was mixed with 0.5 ml of 50 mM potassium phosphate buffer (pH 7.0) containing 2.5 mM of 2-deoxyribose. The reaction was developed at 35 °C in dark for 1 h. After adding 1 ml of 1% TBA in 0.05 M sodium hydroxide and 1 ml of acetic acid, the mixture was boiled for 30 min and immediately cooled on ice. The absorbance of the solution was read at 532 nm [29] and the OH^·^ content was expressed as absorbance units per gram of FW.

### Activities of enzymatic antioxidant scavengers

Extracts for the determination of enzyme activities were prepared under cold conditions [4]. Algal cells were harvested by centrifugation and homogenized in 50 mM phosphate buffer (pH 7.0) containing 1 mM ethylenediaminetetraacetic acid, 0.05% (*v/v*) Triton X-100, 2% (*w/v*) polyvinylpyrrolidone, and 1 mM phenylmethylsulfonyl fluoride. The homogenate was centrifuged at 15,880×*g* for 25 min at 4 °C and the supernatant was used as a crude enzyme extract. The total protein content of the extracts was measured using bovine serum albumin as a standard [30].

The APX (EC 1.11.1.11) activity was determined by the changes in the absorbance at 290 nm due to ascorbate oxidation and calculated using an extinction coefficient of 2.8 mM/cm [31]. One APX unit was defined as the enzyme amount that transforms 1 µmol of ascorbate per minute. The SOD (EC 1.15.1.1) and CAT (EC 1.11.1.6) activities were determined using colorimetric assay kits (Sigma-Aldrich, St. Louis, MO, USA) according to the manufacturer’s instructions.

### Measurement of non-enzymatic antioxidant scavengers

For the determination of proline content, dried microalgal biomass was homogenized with 5 ml of 3% sulphosalicylic acid and filtered through a Whatman filter paper. In a test tube, 1 ml of filtrate was added followed by 1 ml of glacial acetic acid and 1 ml of acid ninhydrin solution. Tubes were incubated in a boiling water bath for 1 h. The reaction was terminated by placing the tubes on ice. Next, 4 ml of toluene was added and the content was vortexed for 20–30 s. The absorbance of the upper pink layer was read at 520 nm. The concentration of proline in the samples was determined from a calibration curve prepared using l-proline as a standard [32].

Total polyphenols in the microalgal cells were determined by a slight modification of the procedure of Chandler and Dodds [33]. Microalgal cells were harvested by centrifugation and homogenized with 5 ml of 80% ethanol using a chilled mortar and pestle. The content was centrifuged at 15,880×*g* for 20 min and the supernatant was collected. The remaining residue was re-extracted, the supernatants were pooled and evaporated to dryness. The residue was dissolved in 5 ml of the distilled water. In a test tube, 1 ml of the aliquot was mixed with 0.5 ml of 1 N Folin–Ciocalteu’s reagent and incubated for 3 min. Then 2 ml of 20% freshly prepared sodium carbonate solution was added to each tube and the content was thoroughly mixed. The solution was incubated at room temperature for 1 h in the dark, and the absorbance was measured at 650 nm. The concentrations of phenols in the samples were calculated from a calibration curve prepared using gallic acid as a standard.

### Endogenous levels of phytohormones ABA and IAA

The extracts for the determination of plant growth regulators (PGRs) were prepared using the extraction buffer [34] containing 2-propanol, concentrated hydrochloric acid, and water (2:0.002:1). The microalgal biomass was harvested by centrifugation and homogenized using extraction buffer under cold condition. The extract was sonicated twice for 5 min (on:off mode of 5:2 s) under the ice-cold condition and further incubated on ice for 15 min. The PGRs were separated from the mixture by the addition of dichloromethane. The mixture was further incubated on ice for 20 min (frequently mixed) and centrifuged at 12,860×*g* for 10 min at 4 °C. The solvent phase from the bottom was transferred to the glass tube and concentrated using a nitrogen steam. The resulting residues were re-dissolved in methanol. The concentrations of ABA and IAA in the extracts were determined using PGR immunoassay detection kits (Sigma-Aldrich, St. Louis, MO, USA) according to the manufacturer’s instructions and expressed on a FW basis.

### Statistical analysis

The experiment was carried out in triplicate, and the data presented are mean values ± standard deviation of three independent replicates. All data were further analyzed by one-way analysis of variance (ANOVA) using InfoStat, 2012. The mean values were compared with the LSD test and a significant difference was considered at *P* < 0.05.

## Results and discussion

### Changes in the biomass production and differences in the cell size of *A. dimorphus* under nitrogen starvation

Nitrogen starvation has been considered as the most prominent technique to enhance the biochemical constituents of microalgae [5]. Although continuous nitrogen limitation increases the lipid and carbohydrate contents of microalgae, it severely decreases their biomass production. Even during the two-stage cultivation, starvation for a longer duration (3, 6 or 9 days) has been found to decrease the biomass production [5], which might result in the lower productivities of lipid and carbohydrate. Therefore, in the present study, *A. dimorphus* was nitrogen-starved for short term of 1, 2, and 3 days through two-stage cultivation. Starvation of 1 day reduced the DCW of *A. dimorphus* from 352 ± 4 mg/l (in control) to 296 ± 7 mg/l. Further increasing the duration of starvation did not significantly change the DCW, i.e., 306 ± 14 and 306 ± 11 mg/l after 2 and 3 days, respectively. Similarly, Sulochana and Arumugam also observed no significant difference in the biomass yield of *Scenedesmus quadricauda* nitrogen-starved for 24, 48, and 72 h [35].

Morphology of the microalgal cells changes depending on their surrounding environment. Nitrogen starvation has been shown to alter the cell size of microalgae. In the present study, changes in the cell size of *A. dimorphus* were observed after transferring the cells to nitrate-free medium. The cell length was doubled under nitrogen-starved conditions. The nitrogen starvation for 1 day increased the cell length from 5.88 ± 0.67 (in control) to 11.93 ± 0.69 µm and cell width from 4.30 ± 0.37 (in control) to 5.22 ± 0.39 µm. Cell lengths of 11.46 ± 0.93 and 11.40 ± 0.92 µm and cell widths of 4.85 ± 0.54 and 5.48 ± 0.74 µm were observed after 2 and 3 days of nitrogen starvation, respectively. In a similar study, Pancha et al. [5] also observed an increase in the cell size of *Scenedesmus* sp. grown in nitrate-starved conditions. Microalga *A. dimorphus* belongs to the *Scenedesmaceae* family, members of which exhibit pleomorphism by changing their cell morphology to produce unicells and coenobia under various environmental conditions [5].

### Changes in the pigments contents and chlorophyll fluorescence of *A. dimorphus* under nitrogen starvation

A chloroplast is a fundamental unit for most of the photosynthetic plants and algae. Therefore, the contents of chlorophylls and carotenoids and the vitality of the photosynthetic apparatus are critical physiological indicators to check the algal cell adaptation during nutrient deficiency [36]. Being a nitrogen-rich compound, chlorophyll is utilized as an intracellular nitrogen pool by the cell for their growth during nitrogen-starved conditions [37]. Carotenoids have been reported to serve a protective function against oxidative stress in microalgae [13]. Therefore, changes in the photosynthetic pigments and chlorophyll fluorescence were measured to study the effects of nitrogen starvation on PS-II activity and thus photosynthesis of *A. dimorphus*. As shown in Table 1, after 3 days of nitrogen starvation, the content of chlorophyll *a* + *b* was 22.96 ± 0.60 mg/g, which was similar to that of the control (23.25 ± 3.14 mg/g). Similarly, there was no significant difference in the carotenoids accumulation by control (4.36 ± 0.83 mg/g) and 3 days nitrogen-starved culture (3.93 ± 0.19 mg/g). This suggests that nitrogen starvation up to 3 days did not significantly affect the photosynthetic pigments contents of *A. dimorphus*.

**Table 1:** The photosynthetic activity of *A. dimorphus* under nitrogen deficiency

Treatments	Chlorophyll *a* (mg/g)	Chlorophyll *b* (mg/g)	Chlorophyll *a* + *b* (mg/g)	Carotenoids (mg/g)	*F*_v_ */F*_m_
Control	16.07 ± 2.33^a^	7.18 ± 0.82^a^	23.25 ± 3.14^a^	4.36 ± 0.83^a^	0.621 ± 0.027^b^
1N-	17.21 ± 1.01^a^	8.03 ± 0.33^a^	25.23 ± 1.16^a^	4.57 ± 0.35^a^	0.674 ± 0.004^a^
2N-	16.93 ± 0.96^a^	8.23 ± 0.83^a^	25.16 ± 1.79^a^	4.55 ± 0.10^a^	0.669 ± 0.005^a^
3N-	14.84 ± 0.53^a^	8.12 ± 0.16^a^	22.96 ± 0.60^a^	3.93 ± 0.19^a^	0.628 ± 0.003^b^

1N-, 2N-, and 3N- represent the cultures with 1, 2, and 3 days of nitrogen starvation, respectively. Values are presented as the mean ± standard deviation (*n* = 3). Values with the different letters represent a significant difference (*P* < 0.05) between treatments

Chlorophyll fluorescence is a widely used technique to measure the photosynthetic performance (mainly PS-II activity) because it is easy, fast, non-invasive, and provides plenty of information about the fundamental performance of the PS-II of the cells under various environmental conditions [38]. The parameter *F*_v_ */F*_m_ represents a maximum potential quantum efficiency of PS-II, if all the reaction centers were open. When microalgae are nutrient limited, the flow of electron from the photosystems to the electron transport chain is impaired, and ROS are formed. This impairment of photosystem is illustrated by the decrease in *F*_v_ */F*_m_ upon nutrient limitation [39] which indicates that PS-II is negatively influenced [40]. In the present study, *F*_v_ */F*_m_ increased marginally (Table 1), but significantly (*P* < 0.05), for the cultures with 1 (0.674 ± 0.004) and 2 (0.669 ± 0.005) days of nitrogen starvation, compared to that of the control culture (0.621 ± 0.027). The 3 days nitrogen-starved cultures exhibited *F*_v_ */F*_m_ of 0.628 ± 0.003, which was similar to that of the control. This result is in accordance with that of the pigments contents, which further confirms that nitrogen starvation up to 3 days did not significantly affect the photosynthetic apparatus of *A. dimorphus*.

### Biochemical composition of *A. dimorphus* under nitrogen starvation

**Fig. 1:**
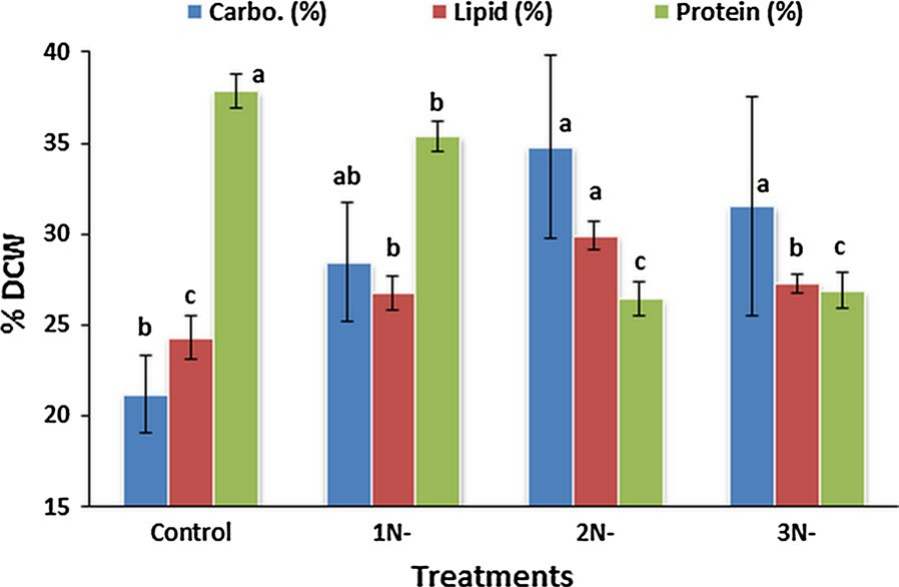
Effects of nitrogen starvation on carbohydrate, lipid, and protein contents of *A. dimorphus*. *1N-*, *2N-*, and *3N-* represent the cultures with 1, 2, and 3 days of nitrogen starvation, respectively. Values are presented as the mean ± standard deviation (*n* = 3). Values with the *different letters* represent a significant difference (*P* < 0.05) between treatments

Carbon fixed from the photosynthesis can be used for the synthesis of major macromolecules like lipids, proteins, and carbohydrates. As nitrogen is required for the synthesis of protein, limited nitrogen concentration affects the synthesis of protein required for the cell division and photosynthesis, which reduces the cell growth rates [41] and thus biomass production. In the present study, the highest protein content of 37.88 ± 0.93% was observed in the control culture with nitrogen sufficient condition (Fig. 1). The protein content reduced significantly (*P* < 0.05) to 35.38 ± 0.83% and then to 26.88 ± 0.98% after 1 and 3 days of nitrogen starvation, respectively.

Nitrogen starvation influences the distribution of carbon in microalgae [42]. During nitrogen sufficient conditions, the molar rate of photosynthetic carbon fixation is 7–10 times higher than the rate of nitrogen assimilation, which is suitable for the synthesis of nitrogen-containing cellular components like proteins, nucleotides, and pigments. During the initial stage of nitrogen deficiency, before photosynthetic capacity is significantly diminished, the carbon fixation exceeds the carbon demands and excess carbon is diverted to storage compounds like lipids and carbohydrates [43, 44] due to their highly reduced states and efficient packing in small compartments of the cells [45]. In the present study, lipid content of *A. dimorphus* was significantly higher (*P* < 0.05) in all three nitrogen starvation treatments compared to that of the control (Fig. 1). It was highest (29.92 ± 0.74%) in 2 days nitrogen-starved cultures, which was about 23% higher than that of the control culture (24.31 ± 1.20%). Under nitrogen deficiency, a significant trigger in the lipid accumulation by various microalgae like *Chlorella vulgaris* [2, 46], *Scenedesmus* sp. [5], *Dunaliella salina* [12], *Chlorella sorokiniana* [13], *Nannochloropsis oceanica* [47] is reported previously. Considering the changes in the chlorophyll fluorescence, Zhang et al. concluded that prolonged nitrogen starvation in *C. sorokiniana* increases cyclic electron flow around PSI, which supplies ATP to TAG synthesis [13]. The possible reason behind a trigger in the lipid accumulation under nitrogen deficiency might be degradation of the nitrogenous compounds like chlorophyll and protein, which might provide carbon or energy to the cell for the accumulation of lipid [2, 5, 44, 48, 49]. In our results, although there were no significant changes in the chlorophyll contents, the protein content of the cells decreased significantly (*P* < 0.05) up to 2 days of nitrogen starvation (Fig. 1), thus supporting the above perception. Extending the nitrogen starvation for 3 days did not further decrease the protein content of the cells. Moreover, there was no significant difference in the carbohydrate contents of the cells nitrogen-starved for 2 and 3 days. This might have forced the cells to degrade their lipid, the energy-rich compounds, to meet the energy demand and cope with with the acute stress conditions after 2 days of nitrogen starvation. It was further confirmed by the fractionation of total lipid as discussed below.

**Fig. 2:**
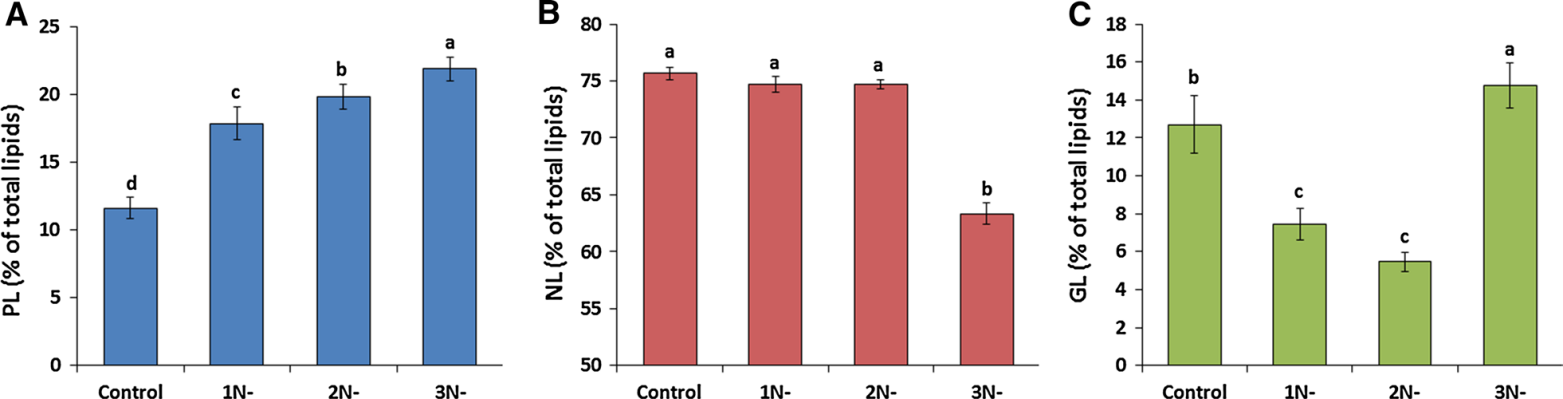
Variations in the lipid class composition of *A. dimorphus* under nitrogen deficiency. **A** Phospholipids (PL), **B** neutral lipids (NL), and **C** glycolipids (GL). *1N-*, *2N-*, and *3N-* represent the cultures with 1, 2, and 3 days of nitrogen starvation, respectively. Values are presented as the mean ± standard deviation (*n* = 3). Values with the *different letters* represent a significant difference (*P* < 0.05) between treatments

Microalgal lipid is composed of NLs, GLs, and PLs. NLs are mainly composed of TAGs, which is important for biodiesel production. The PLs and GLs are important components of the external membrane and the membranes of chloroplast and endoplasmic reticulum [5]. To find out the effects of nitrogen starvation on the lipid composition of *A. dimorphus*, total lipid was further fractionated by a column chromatography. As shown in Fig. 2, PLs of the cells increased significantly (*P* < 0.05) with the increase in the duration of nitrogen starvation, i.e., 11.62 ± 0.80% in control to 21.90 ± 0.90% in 3 days nitrogen-starved culture. Up to 2 days of nitrogen starvation, the accumulation of NLs was maintained in the cells (74.67 ± 0.40%). The GLs decreased significantly (*P* < 0.05) from 12.70 ± 1.50% in control to 5.46 ± 0.50% in 2 days nitrogen-starved culture. Extending the nitrogen starvation for 3 days significantly (*P* < 0.05) reduced the neutral lipids to 63.33 ± 0.95% and increased the glycolipids to 14.76 ± 1.20%. This result is different from those of Pancha et al. who observed a significant increase in the neutral lipid content of *Scenedesmus* sp. from 67.56 to 81.81% after 3 days of nitrogen starvation [5]. These results suggest that cells undergo membrane remodeling to combat the stress conditions generated during nitrogen deficiency.

Microalgal carbohydrates are considered as a potential resource for bioethanol production due to their advantages like easy conversion to fermentable sugars, no harsh pretreatment, and easy hydrolysis due to the absence of lignin [50]. Similar to lipid accumulation, the carbohydrate content of *A. dimorphus* also increased with the increase in the duration of nitrogen starvation (Fig. 1). It was maximum (34.80 ± 5.03%) after 2 days of nitrogen starvation, which was about 64% higher than that of the control culture (21.21 ± 2.12%). Further increasing the duration of nitrogen starvation to 3 days did not significantly affect the accumulation of carbohydrate. Previously, Jia et al. reported a prominent increase in the cellular carbohydrate content of *N. oceanica* from 5.8 to 17.9% in the 4 days nitrogen-starved culture [47], while Pancha et al. did not observe any significant difference in the carbohydrate accumulation by *Scenedesmus* sp. nitrogen-starved for 3 days [5]. Our results are in accordance with the renowned perception that the biosynthesis of carbohydrate is dominant over lipid accumulation in microalgae and higher synthesis of TAGs occurs only when carbon supply exceeds the capacity of starch synthesis [51]. From the biochemical composition of *A. dimorphus*, it could be inferred that nitrogen starvation for a short term of 2 days should be preferable to produce biomass with higher lipid and carbohydrate contents.

### ROS contents and responses of cellular antioxidants of *A. dimorphus* under nitrogen starvation

**Fig. 3:**
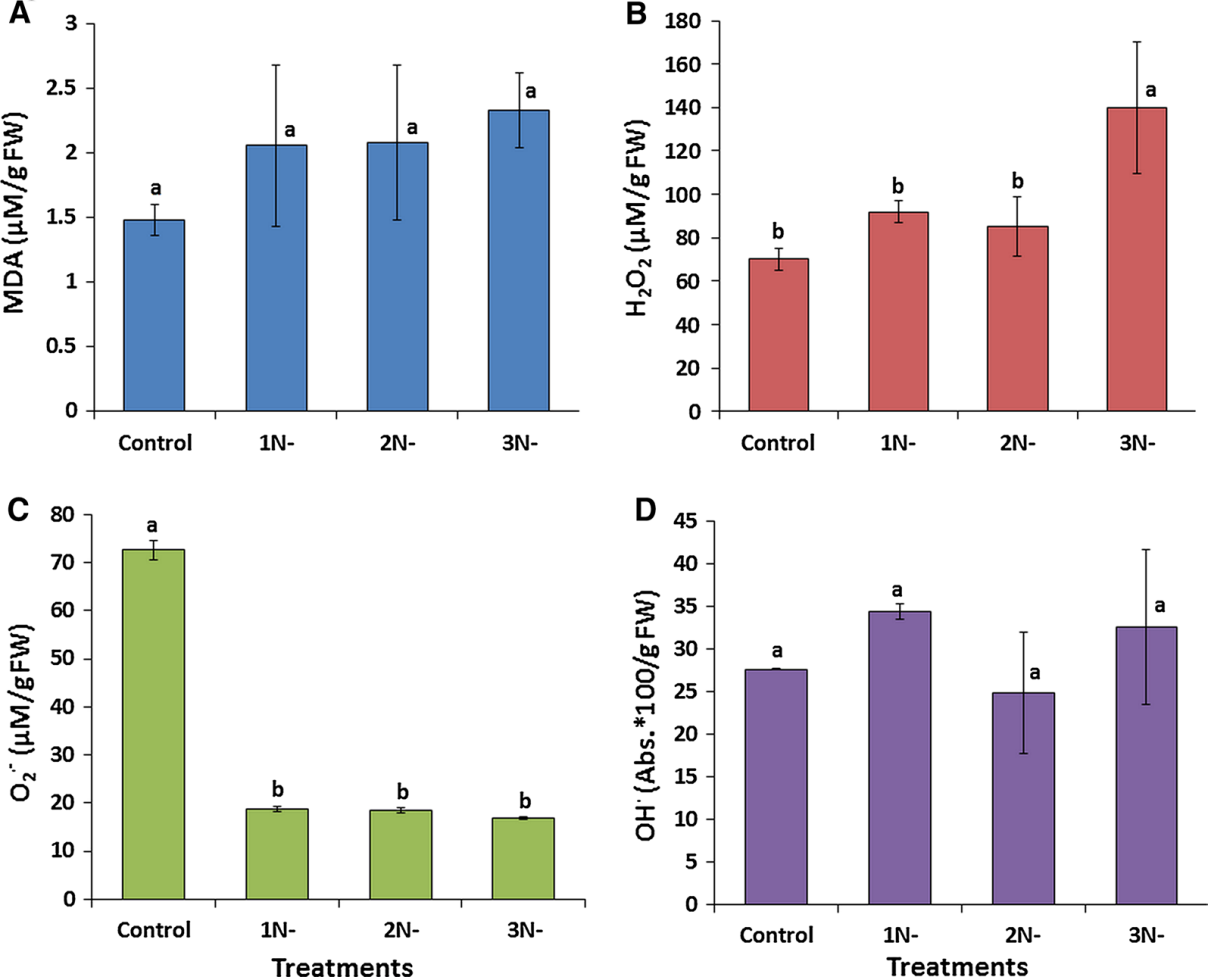
Effects of nitrogen starvation on **A** malondialdehyde (MDA), **B** hydrogen peroxide (H_2_O_2_), **C** superoxide radicals (O_2_^·−^), and **D** hydroxyl radicals (OH^·^) contents in *A. dimorphus*. *1N-*, *2N-*, and *3N-* represent the cultures with 1, 2, and 3 days of nitrogen starvation, respectively. Values are presented as the mean ± standard deviation (*n* = 3). Values with the *different letters* represent a significant difference (*P* < 0.05) between treatments

Algal cells activate several defense systems for scavenging the ROS generated in different cellular compartments. Under unfavorable conditions, the generation rate of ROS exceeds their scavenging rate, and the excess ROS cause oxidative injury to the cell [52]. In the present study, we investigated the accumulation of three ROS (H_2_O_2_, O_2_^·−^, and OH^·^) along with the level of lipid peroxidation in terms of MDA content (Fig. 3), the responses of cellular enzymatic antioxidants (SOD, CAT, and APX) and non-enzymatic scavengers (proline and total polyphenols) to further understand and link their roles in the accumulation of lipid and carbohydrate by *A. dimorphus* under oxidative stress conditions generated during nitrogen deficiency.

The H_2_O_2_ content of the cells (Fig. 3B) did not change significantly up to 2 days of nitrogen-starved conditions (85.07 ± 13.54 µM/g FW) and then, after 3 days of starvation, increased significantly (*P* < 0.05) by twofolds (139.93 ± 30.21 µM/g FW) in comparison to that of the control culture (70.05 ± 5.23 µM/g FW). The O_2_^·−^ content (Fig. 3C) in 1-day nitrogen-starved cultures decreased significantly (*P* < 0.05) by almost fourfolds (18.73 ± 0.57 µM/g FW) than that of the control culture (72.64 ± 2.12 µM/g FW) and then remained statistically similar (*P* < 0.05) up to 3 days of nitrogen starvation. Although the content of OH^·^ did not vary significantly in all four treatments (Fig. 3D), it was numerically lowest (24.85 ± 7.12 absorbance * 100/g FW) in the 2 days nitrogen-starved cultures. The varying levels of ROS in all three nitrogen-starved cultures suggest a difference in the degree of a cellular defense response. Similar to the OH^·^ content, the MDA content also did not vary significantly in all four treatments (Fig. 3A). This was mainly due to a larger margin of errors in 1 and 2 days nitrogen-starved cultures. However, the MDA content was numerically higher (2.33 ± 0.29 µM/g FW) in 3 days nitrogen-starved culture. From these results, it can be understood that the cell growth exhibits an inverse relation with that of the MDA content and ROS levels, particularly H_2_O_2_, which indicates that the growth inhibition might be caused by a high ROS toxicity generated during nitrogen deficiency. Similar to our study, Yilancioglu et al. also observed an increased production of ROS as well as higher lipid peroxidation in *D. salina* under nitrogen-limited conditions [12]. Fan et al. reported an increase in the contents of OH^·^ and MDA in *Chlorella pyrenoidosa* under nitrogen deficiency [36]. Zhang et al. also observed a significant increase in the MDA level of *C. sorokiniana* during oil droplet formation caused by the nitrogen starvation-induced oxidative stress [13].

To counter the ROS toxicity, cells employ the innate defense responses. This includes an array of the enzymatic antioxidants like SOD, CAT, APX, and various non-enzymatic scavengers like proline, polyphenols [9, 52]. Among enzymatic antioxidants, SOD provides the first line of defense against toxic effects of ROS by catalyzing the dismutation of O_2_^·−^ (two superoxide radicals are neutralized by the addition of two hydrogen ions) to H_2_O_2_ and O_2_, thereby decreasing the risk of OH^·^ formation via Haber–Weiss-type reaction. CAT contains porphyrin heme active sites that directly dismutate H_2_O_2_ to H_2_O and O_2_. APX is one of the peroxidases involved in the scavenging of H_2_O_2_ utilizing ascorbate as the electron donor [52]. The increase in the activities of antioxidant enzymes SOD, CAT, peroxidases, glutathione reductase, etc., has been reported previously in the microalgae grown under a different nutrient limitation or starvation conditions including nitrogen starvation [12, 13, 36, 53, 54]. In the present study, the activity of SOD increased in all three starvation treatments, compared to that of the control (Table 2). In the 2 days nitrogen-starved culture, it was over fivefolds higher (3857.92 ± 1052.67 U/mg protein) than that of the control culture (687.26 ± 222.87 U/mg protein). In the 3 days nitrogen-starved culture, the activity of SOD decreased by threefolds (1273.73 ± 155.25 U/mg protein) than that of the 2 days nitrogen-starved culture; still, it was about 1.85-folds higher than that of the control culture. The CAT activity showed continuous and significant (*P* < 0.05) increase in all three starvation treatments exhibiting a corresponding relevance with the H_2_O_2_ contents. The highest CAT activity (54.07 ± 2.79 × 10^3^ U/mg protein) was observed in the 3 days nitrogen-starved culture, which was over twofolds higher than that of the control culture (24.72 ± 1.17 × 10^3^ U/mg protein). Contrarily, the APX activity decreased in all three nitrogen-starved cultures compared to that of the control culture (6.60 ± 0.28 U/mg protein). This might be due to the predominance of CAT over APX, as the CAT is thought to contribute more strongly than the APX to the elimination of H_2_O_2_ [55]. CAT has one of the highest turnover rates, i.e., in 1 min, one molecule of CAT can convert approximately 6 million molecules of H_2_O_2_ to H_2_O and O_2_ [52]. As CAT activity highly increased in the nitrogen-starved cultures, the availability of H_2_O_2_ might be lower for the APX, ultimately reducing its activity. Another possible reason behind reduced APX activities in the nitrogen-starved cultures might be the degradation of ascorbate, a substrate required by the APX to scavenge H_2_O_2_ in water–water and ascorbic acid–glutathione cycles [52], under nitrogen deficiency.

**Table 2:** Activities of the enzymatic antioxidants of *A. dimorphus* under nitrogen deficiency

Treatments	SOD (U/mg protein)	CAT (U × 10^3^/mg protein)	APX (U/mg protein)
Control	687.26 ± 222.87^d^	24.72 ± 1.17^c^	6.60 ± 0.28^a^
1N-	1682.15 ± 70.63^b^	24.72 ± 0.40^c^	2.28 ± 0.30^bc^
2N-	3857.92 ± 1052.67^a^	38.63 ± 0.63^b^	2.81 ± 0.11^b^
3N-	1273.73 ± 155.25^c^	54.07 ± 2.79^a^	2.06 ± 0.42^c^

1N-, 2N-, and 3N- represent the cultures with 1, 2, and 3 days of nitrogen starvation, respectively. Values are presented as the mean ± standard deviation (*n* = 3). Values with the different letters represent a significant difference (*P* < 0.05) between treatments

**Fig. 4:**
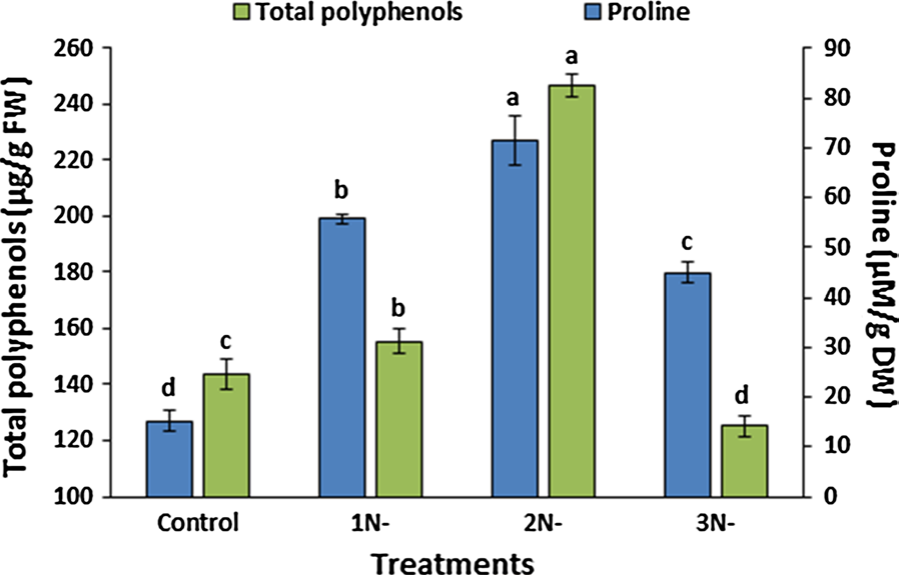
Accumulations of proline and total polyphenols by *A. dimorphus* under nitrogen deficiency. *1N-*, *2N-*, and *3N-* represent the cultures with 1, 2, and 3 days of nitrogen starvation, respectively. Values are presented as the mean ± standard deviation (*n* = 3). Values with the *different letters* represent a significant difference (*P* < 0.05) between treatments

Among the non-enzymatic antioxidants, the levels of proline and total polyphenols were measured in the present study. Proline acts as an antioxidant by being a scavenger of OH^·^ and ^1^O_2_ [56, 57]. In the present study, the accumulation of proline was significantly higher (*P* < 0.05) in all three nitrogen-starved cultures in comparison to that in control culture (Fig. 4). It was highest in the 2 days nitrogen-starved culture (71.52 ± 4.83 µM/g DW), which was about 4.7-folds higher than that of the control culture (15.14 ± 2.14 µM/g DW). The culture with 3 days of nitrogen starvation exhibited a significant reduction in the accumulation of proline, i.e., 44.90 ± 2.01 µM/g DW, compared to other two starvation treatments; still, it was about threefolds higher than that of the control culture. This result is different from that of Pancha et al. [5], who observed a significant reduction in the proline content of *Scenedesmus* sp. (twofolds over control) after 3 days of nitrogen starvation, thus suggesting the difference in the strategy of different microalgae to cope with the nitrogen starvation-induced oxidative stress conditions.

Polyphenols act as the substrates for the H_2_O_2_-scavenging enzyme peroxidase and prevent the diffusion of ROS by altering the peroxidation kinetics and reducing the fluidity of the cell membrane [58]. The pattern of accumulation of total polyphenols was identical to that of the proline. The highest accumulation of total polyphenols was observed in the 2 days nitrogen-starved culture, i.e., 246.75 ± 4.19 µg/g FW, which was about 1.7-folds higher than that of the control culture (143.47 ± 5.47 µg/g FW). Despite the promoted antioxidant properties of the polyphenols and the potential of microalgae as a source of polyphenols, there are limited studies on the accumulation of polyphenols by microalgae [9]. Goiris et al. reported a reduction in the phenolics content of three microalgal strains *Chlorella*, *Phaeodactylum*, and *Tetraselmis* in nitrogen-limiting treatments [39]. After UV exposure, Kovacik et al. observed no change in the phenolics content of *S. quadricauda* [59], while Duval et al. observed an increase in the phenolics content of *Chlamydomonas nivalis* [60]. A great deal of more research is required to determine how polyphenols respond to the accumulation of ROS in different microalgae under different stress conditions.

### Endogenous levels of phytohormones in *A. dimorphus* under nitrogen starvation

The increasing evidence suggests that the pathways of phytohormones are important in mediating the oxidative stress signaling [61] as phytohormones are important signaling compounds involved in the plant defenses [62]. As ROS and phytohormones could interact with each other and fine tune the plant defenses, the intracellular levels of phytohormones ABA and IAA were detected in the present study. ABA is a well-known stress hormone in higher plants that becomes active during adaptation to various abiotic stresses [55]. IAA is the most abundant and naturally occurring auxin, which regulates the growth and development of higher plants [63]. These phytohormones are expected to play a homologous role in microalgae, as algae are phylogenetically related to the plants [64]. While treatments with the exogenous addition of various phytohormones have been shown to stimulate the growth and stress tolerance of microalgae [65–67], very limited data are available on their endogenous levels in microalgae under stress conditions [68].

**Fig. 5:**
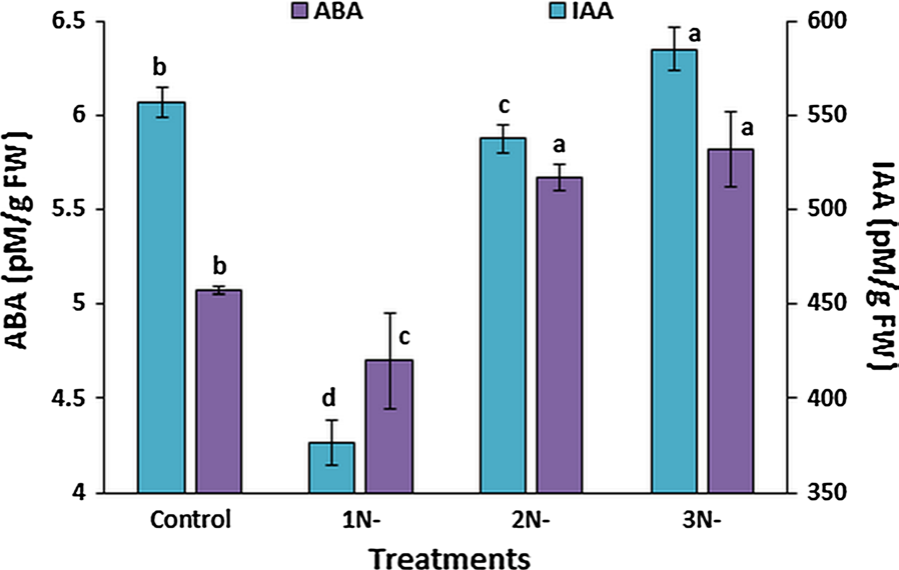
The endogenous levels of phytohormones abscisic acid (ABA) and indole-3-acetic acid (IAA) in *A. dimorphus* under nitrogen deficiency. *1N-*, *2N-*, and *3N-* represent the cultures with 1, 2, and 3 days of nitrogen starvation, respectively. Values are presented as the mean ± standard deviation (*n* = 3). Values with the *different letters* represent a significant difference (*P* < 0.05) between treatments

In the present study, nitrogen starvation for 1 day significantly (*P* < 0.05) decreased the synthesis of ABA, i.e., 5.07 ± 0.02 pM/g FW in control to 4.70 ± 0.26 pM/g FW (Fig. 5). However, extending the duration of nitrogen starvation resulted in significant (*P* < 0.05) increase in the synthesis of ABA, which was highest (5.82 ± 0.20 pM/g FW) in the 3 days nitrogen-starved culture. The pattern of IAA synthesis was identical to that of the ABA synthesis. Nitrogen starvation of 1 day decreased the IAA level by 0.67-fold (376.40 ± 11.80 pM/g FW) compared to that of the control (556.83 ± 7.72 pM/g FW). Extending the nitrogen starvation to 3 days significantly (*P* < 0.05) increased the synthesis of IAA, which was about 1.55-folds higher (585.08 ± 11.75 pM/g FW) than that of the 1-day nitrogen-starved culture. These results show that the nitrogen starvation-induced oxidative stress triggered stimulation of the biosynthesis pathways of ABA and IAA. Similar to our study, Lu et al. also observed transcriptional upregulation of the biosynthetic pathways of ABA in the nitrogen-deficient oleaginous microalga *N. oceanica* [68] suggesting the role of ABA in the alleviation of the stress damage. Sulochana and Arumugam [35] also observed an immediate increase (fourfolds over control) in the ABA level of *S. quadricauda* during the onset of 24 h of nitrogen deprivation; however, the ABA level further decreased when the nitrogen deprivation was continued up to 72 h.

It has been reported that ABA exhibits a protective effect on the photosystem II complex in *Chlamydomonas reinhardtii* by accelerating its recovery from the photo-induced inactivation occurring under low temperature [69] through ROS-eliminating antioxidant enzymes SOD and CAT [55]. In the present study, the accumulation of ABA once decreased after 1 day of nitrogen starvation, and increased significantly (*P* < 0.05) after 2 days, which was maintained by the cells even after 3 days of starvation. This can be linked with the significantly higher activities of the ROS-scavenging enzymatic antioxidants SOD and CAT after 2 days of nitrogen starvation.

## Conclusion

In the present study, we made an investigation into the cell growth, physiology, and the biofuel potential of oleaginous green microalga *A. dimorphus* through short-term nitrogen starvation. We also made an attempt to link the accumulation of lipid and carbohydrate with the oxidative stress, the response of cellular antioxidants, and the endogenous levels of phytohormones under nitrogen deficiency.

The findings of the present study highlight the interaction of nitrogen starvation-induced oxidative stress with the signaling involved in the growth and development of *A. dimorphus*. The contents of lipid and carbohydrate were highest in 2 days nitrogen-starved culture. In the cell, O_2_^·−^ is either directly catalyzed to OH^·^ via Haber–Weiss cycle or to H_2_O_2_, by the action of SOD, which through the Fenton reaction produces OH^·^. Although the levels of O_2_^·−^ were similar in all three nitrogen-starved cultures, there was a significant difference in the accumulation of H_2_O_2_ and OH^·^. The levels of both these ROS were lower in 2 days nitrogen-starved culture, compared to those in 1 and 3 days nitrogen-starved cultures. This can be linked with the powerful antioxidant defense in 2 days nitrogen-starved culture as shown by the higher activities of SOD and CAT, the highest accumulation of proline, total polyphenols, and the stress hormone ABA which is reported to be active during adaptation to various abiotic stresses.

This study, thus, provides the strategy to improve the biofuel potential of *A. dimorphus* through a short-term nitrogen starvation along with presenting a comprehensive picture of the adaptive mechanisms of the cells from a physiological perspective.

## Abbreviations

ABA: abscisic acid
ANOVA: analysis of variance
APX: ascorbate peroxidase
CAT: catalase
DCW: dry cell weight
DW: dry weight
*F*_v_/*F*_m_: maximum quantum yield of photosystem II
FW: fresh weight
GL: glycolipid
H_2_O_2_: hydrogen peroxide
IAA: indole-3-acetic acid
MDA: malondialdehyde
NL: neutral lipid
O_2_^·−^: superoxide radicals
OH^·^: hydroxyl radicals
PAM: pulse amplitude modulated
PGRs: plant growth regulators
Phytohormones: plant hormones
PL: phospholipid
PS II: photosystem II
ROS: reactive oxygen species
SOD: superoxide dismutase
TBA: thiobarbituric acid
TCA: trichloroacetic acid
